# A Technical–Economic Study on Optimizing FDM Parameters to Manufacture Pieces Using Recycled PETG and ASA Materials in the Context of the Circular Economy Transition

**DOI:** 10.3390/polym17010122

**Published:** 2025-01-06

**Authors:** Dragos Gabriel Zisopol, Mihail Minescu, Dragos Valentin Iacob

**Affiliations:** 1 Mechanical Engineering Department, Petroleum-Gas University of Ploiesti, 100680 Ploiesti, Romania; mminescu@upg-ploiesti.ro; 2 Department of Mechanical Engineering, Doctoral School, Petroleum-Gas University of Ploiesti, 100680 Ploiesti, Romania

**Keywords:** 3D printing, FDM, value analysis, printing parameters, optimization, rPETG, rASA, tensile, compressive

## Abstract

This paper presents the results of research on the technical–economic optimization of FDM parameters (L_h_—layer height and I_d_—infill density percentage) for the manufacture of tensile and compression samples from recycled materials (r) of PETG (polyethylene terephthalate glycol) and ASA (acrylonitrile styrene acrylate) in the context of the transition to a circular economy. To carry out our technical–economic study, the fundamental principle of value analysis was used, which consists of maximizing the ratio between *V_i_* and *C_p_*, where *V_i_* represents the mechanical characteristic (tensile strength or compressive strength) and *C_p_* represents the production cost. The results of this study showed that, in the case of tensile samples manufactured by recycled PETG (rPETG), the parameter that significantly influences the results of the *V_i_/C_p_* ratios is L_h_ (the height of the layer), while for the samples manufactured additively from recycled ASA (rASA), the parameter that decisively influences the tensile strength is I_d_ (the infill density percentage). In the case of compression samples manufactured by FDM from recycled PETG (rPETG) and recycled ASA (rASA), the parameter that signified influences the results of the *V_i_/C_p_* ratios is I_d_ (the infill density percentage). Following the optimization of the FDM parameters, using multiple-response optimization, we identified the optimal parameters for the manufacture of parts by FDM from rPETG and rASA: L_h_ = 0.20 mm and I_d_ = 100%. The results of this study demonstrated that the use of recycled plastics from PETG and ASA lends itself to a production and consumption model based on a circular economy.

## 1. Introduction

In the current economic context, reducing production costs is essential for achieving companies’ financial objectives, and to achieve these goals, optimizing manufacturing processes plays a major role [1,2,3,4,5,6,7,8]. At the same time, finding solutions for efficient waste management is a global challenge [9,10,11,12,13,14,15,16,17,18,19]. Additive manufacturing technologies stand out globally due to the following advantages they have compared to conventional manufacturing technologies: low operating costs, simplicity of use, and efficiency of material use [20,21,22,23,24,25,26,27,28,29,30,31]. The concept of production and consumption based on a circular economy promotes sustainability by increasing the lifespan of materials, as they are recovered and reused, thus reducing the amount of waste, the amount of energy required for the manufacture of materials, and production costs [32,33,34,35,36,37,38,39,40,41,42]. There is significant interest in the research on implementing the circular economy-based production and consumption model in the field of additive manufacturing technologies through thermoplastic extrusion; however, studies focused on recycled materials made from PETG (polyethylene terephthalate glycol) and ASA (acrylonitrile styrene acrylate) are limited.

In [37], an experimental study on the optimization of FDM parameters (layer height: 0.18, 0.25, and 0.33 mm; filling pattern: solid, sparse double dense, and hexagonal; orientation angle: 0, 45, and 90 degrees; printing plane: XY, XZ, and YZ; part position: 1, 5, and 9) was presented with the aim of reducing the post-processing time and energy consumption without affecting the dimensional accuracy of the additively manufactured parts from ASA. The optimization of the FDM parameters resulted in the following optimal solutions: layer height: 0.33 mm; filling pattern: sparse double dense; orientation angle: 90 degrees; printing plane: XY; part position: 9.

DePalma et al. [43] presented the results of research on the evaluation of the use of additive manufacturing technologies by the extrusion of plastics (FDM) and selective laser sintering (SLS) in the context of the circular economy. The conclusions of the study showed that in the case of SLS, by reusing PA12 (polyamide 12), the cost of a part can be reduced by 10%, and in the case of FDM, by reusing ABS, the cost of a part can be reduced by up to 80%. At the same time, the study presented the problem of polymer degradation during the printing cycle due to the reuse of materials.

The authors of [44] presented an investigation into the ecological and economic impacts of additive manufacturing technologies on supply chains in the case of tire production. The conclusions of the study showed that the implementation of additive manufacturing technologies in the production process led to an increase in the company’s profitability, production costs were more efficient by 51–61%, and the amount of carbon emissions was reduced by 9–10%.

In [45], a study on the optimization of FDM parameters for the manufacture of PLA lattice structures was presented. To carry out the study, compression specimens were manufactured by FDM according to the ASTM D695 standard; subsequently, all specimens were tested in compression using a speed of 2 mm/min. The optimization of the parameters was performed using the Taguchi method. The conclusions of the study showed that the maximum modulus of elasticity was obtained for the specimens manufactured with the following parameters: layer height, L_h_ = 0.10 mm; extruder temperature, E_t_ = 205 °C; printing speed, P_s_ = 50 mm/s; platform temperature, P_t_ = 60 °C. The results of the ANOVA (analysis of variance) showed that the parameter that decisively influences the compressive strength (C_s_) is L_h_ (layer height).

In [46], a study on the optimization of FDM printing parameters for the manufacture of parts from PLA (polylactic acid) reinforced with CGF (continuous glass fiber) was presented. The conclusions of the study showed that the maximum percentage of impregnation with CGF is 45%, and the optimal parameters of FDM for the manufacture of parts from polylactic acid (PLA) reinforced with continuous glass fiber (CGF) are as follows: nozzle diameter, N_d_ = 1.20 mm; printing speed, P_s_ = 4 mm/s; and extruder temperature, E_t_ = 210 °C.

In [47], a study on the optimization of FDM parameters was presented [extrusion temperature T_e_ = (190; 210; 230) °C, layer height L_h_ = (0.10; 0.20; 0.30) mm; print speed, P_s_ = (40; 60; 80) mm/s; infill density, I_d_ = (30; 60; 90)%], with the aim of maximizing the mechanical characteristics as well as the quality characteristics of the parts manufactured from PLA. The research results showed that the optimal parameters of FDM were as follows: T_e_ = 220 °C; L_h_ = 0.10 mm; P_s_ = 60 mm/s; and I_d_ = 90%.

In [48], a study on the optimization of the manufacturing parameters of FDM (extrusion temperature T_e_; print speed P_s_; layer height L_h_) was presented in order to minimize the energy consumption required for the manufacture of parts from rPET and PLA without affecting the mechanical characteristics. For the manufacture of tensile specimens from PLA, the following manufacturing parameters were used: T_e_ = (180; 200; 220) °C; P_s_ = (30; 50; 60; 70) mm/s; L_h_ = (0.10; 0.20; 0.30) mm. After optimizing the parameters, the following settings were presented: Te = 200 °C; Ps = 60 mm/s; Lh = 0.20 mm. The manufacture of rPET tensile specimens was carried out with the following parameters: T_e_ = (220; 240; 260) °C; P_s_ = (30; 50; 60; 70) mm/s; L_h_ = (0.10; 0.20; 0.30) mm. After optimizing the parameters, the following settings were presented: T_e_ = 240 °C, P_s_ = 60 mm/s, L_h_ = 0.20 mm. The results of this study demonstrated that recycled materials can be used in additive technology applications by extruding plastics without affecting the mechanical characteristics. At the same time, this study showed that printing parameters affect the energy consumption of the FDM printer, as well as the characteristics of the parts.

This paper presents the results of the technical–economic study on the optimization of FDM parameters (L_h_—layer height and I_d_—infill density percentage) for the manufacturing of tensile and compression samples using recycled materials (r) from PETG and ASA. The uniqueness of this research lies in the utilization of the value analysis (AV) principle, which aims to maximize the values of the ratios of the value in use (V_i_) to the production cost (C_p_). This study also highlights the opportunities for using recycled materials in the context of the transition to a circular economy.

## 2. Materials and Methods

The parameters used for manufacturing tensile and compression samples by FDM include the following: part orientation (P_o_); extruder temperature (E_t_); platform temperature (P_t_); printing speed (P_s_); infill pattern (I_p_); layer height (L_h_); infill density (I_d_); and plate adhesion (P_a_).

The variable parameters of FDM used in the manufacture of tensile and compression samples from rPETG and rASA include the following: the layer height, L_h_ = (0.10; 0.15; 0.20) mm, and the infill density percentage, I_d_ = (50; 75; 100) %. The mechanical properties of tensile and compression samples manufactured from rPETG were previously determined by the authors in the works [38,39]; the mechanical properties of compression samples manufactured from rASA were determined by the authors in the work [40], and the results of the breaking strengths of tensile samples manufactured additively from rPETG are presented in this work.

Using the FDM parameters summarized in Table 1, 54 tensile samples (27 of rPETG and 27 of rASA) were fabricated on the Anycubic Pro Max 2.0 3D printer (Shenzhen, China) according to ASTM D638-14 [49] and 90 compression samples (45 of rPETG and 45 of rASA) according to ISO 604:2002 [50]. All 54 tensile samples and 90 compression samples fabricated from Everfil rPETG and rASA filaments on the 3D printer Anycubic Pro Max 2.0 3D were tested by the authors on the Barrus White 20 kN universal testing machine (Budapest, Hungary) in the laboratories of the Faculty of Mechanical and Electrical Engineering of the Petroleum–Gas University of Ploiesti.

Table 1 presents the FDM printing parameters used to manufacture tensile and compressive samples from rPETG and rASA [38,39,40].

Using the results obtained from the experimental determinations of tensile and compressive strength, the production cost for each set of samples and the fundamental principle of value analysis, presented in Relation (1), a technical–economic study on the optimization of FDM parameters for the manufacture of rPETG and rASA samples was conducted. The fundamental principle of value analysis involved in maximizing the ratio between the value in use (V_i_) and the production cost (C_p_) [41,42].

(1) $$\frac{V_{i}}{C_{p}}\to max$$

where V_i_ represents the use value (mechanical characteristic), and C_p_ represents the production cost expressed in monetary units.

The optimization of the ratio between V_i_ and C_p_ was performed using Minitab 19 software.

The production cost calculation of tensile and compression samples produced through FDM using rPETG and rASA was done utilizing Relation (2).

(2) $$C_{p}=Q_{mat}\times P_{mat}+P_{t}\times E_{c}\times P_{en}$$

where $C_{p}$ represents the production cost (EUR); $C_{mat}$ represents the material cost (EUR); $C_{en}$ represents the energy cost (EUR); $Q_{mat}$ represents the material quantity (g); $P_{mat}\;$ represents teh material price (EUR/g); $P_{t}$ represents the printing time (h); $E_{c}$ represents the energy consumption (kW); and $P_{en}$ represents electricity price (EUR/kWh).

The following constant values were used for economic calculations: $P_{m}$ = 0.019 EUR/g (for rPETG); $P_{m}$ = 0.020 EUR/g (for rASA); $P_{en}$ = 0.25 kW/h; and $E_{c}$ = 0.23 kW/h [51,52]. The corresponding values for material consumption and printing time for each set of samples were generated using Cura Slicer software.

Table 2 shows the dimensions and test conditions for the tensile and compression samples, as referenced in [38,39,40,49,50].

## 3. Results and Discussion

### 3.1. Applications of Value Analysis for Analyzing the Mechanical Behavior of rPETG and rASA 3D-Printed Samples

#### 3.1.1. Tensile Testing

Table 3 and Table 4 summarize the costs associated with materials (C_mat_) and electrical energy (C_en_), as well as the overall production cost (C_p_).

The FDM parameters considered (L_h_—layer height and I_d_—infill density percentage) influence the tensile strength of samples manufactured from rPETG and rASA, as well as the electrical energy consumption [41,48]. The results of the V_i_/C_p_ ratio are presented in Table 5 and Table 6.

**Figure 1:**
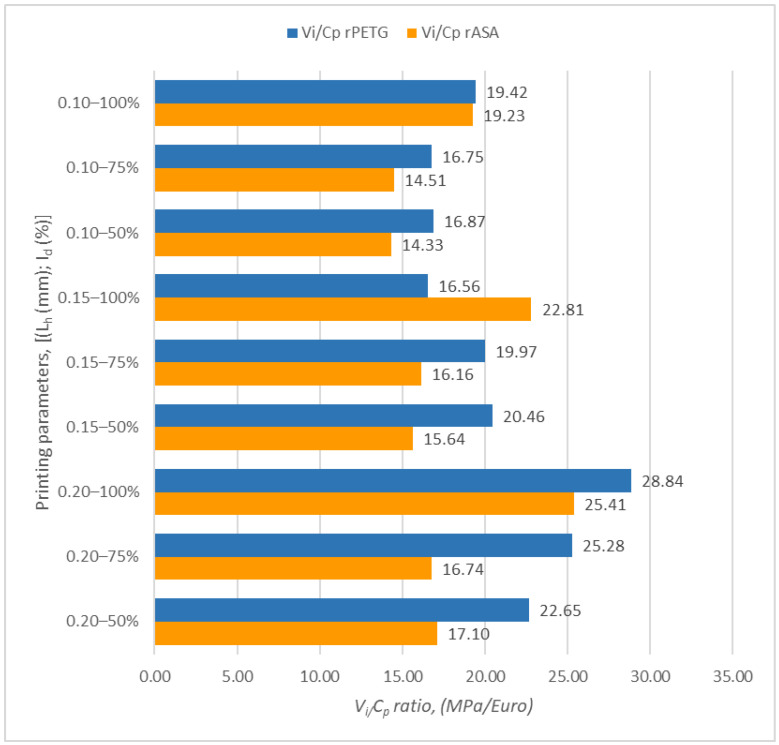
Ratio determination *V_i_/C_p_* for tensile samples made from rPETG and rASA.

Figure 1 shows the charts illustrating the outcomes of the ratios between V_i_ and C_p_ of samples produced through FDM using filament from rPETG and rASA.

Upon examining Figure 1, it can be seen that the highest value (28.84 MPa/EUR) for V_i_ (tensile strength) relative to C_p_ (cost of production) was achieved for the samples created from rPETG, with a layer height of 0.20 mm and an infill density of 100%. For the samples produced from rASA, the peak value of the ratio between V_i_ and C_p_ was also obtained for the additively manufactured samples, which had a layer height of 0.20 mm and an infill density of 100%.

By comparing the minimum with the maximum result of the *V_i_*/*C_p_* ratios obtained for the samples manufactured from rPETG and those corresponding to the rASA samples, it was observed that the *V_i_*/*C*_p_ ratios of the rPETG samples are superior by a percentage range between 11.87 and 13.49% compared to those obtained for the rASA samples.

**Figure 2:**
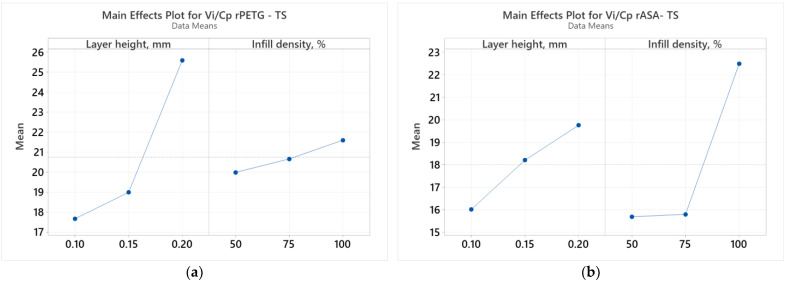
Main effects plots for tensile strength: (**a**) rPETG; (**b**) rASA.

Figure 2 illustrates the relationships between the variable parameters of FDM (L_h_ and I_d_) and the ratio between tensile strength (V_i_) and cost of production (C_p_) [41].

**Figure 3:**
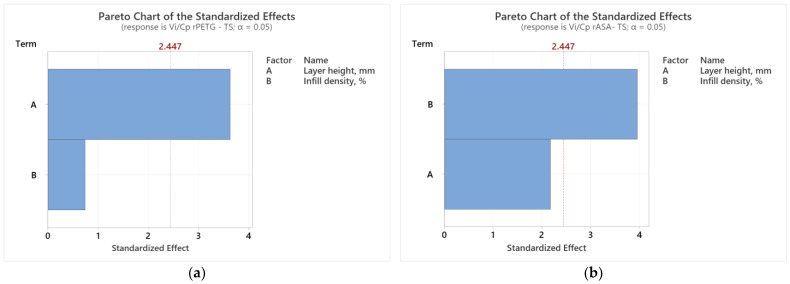
Pareto charts for tensile strength: (**a**) rPETG; (**b**) rASA.

Figure 2 shows how the variable parameters (L_h_ and I_d_) affect the results of the *V_i_/C_p_* ratios for tensile samples made from rPETG (Figure 3a) and rASA (Figure 3b). In Figure 3a, the layer height (L_h_) is the FDM parameter that decisively influences the results of the *V_i_/C_p_* ratios of the tensile samples produced from rPETG. In Figure 3b, we observe that the infill density percentage (I_d_) is the FDM variable parameter that has a significant influence on the results of the *V_i_/C_p_* ratios of the tensile samples manufactured from rASA. These conclusions are supported by the Pareto charts presented in Figure 3.

**Figure 4:**
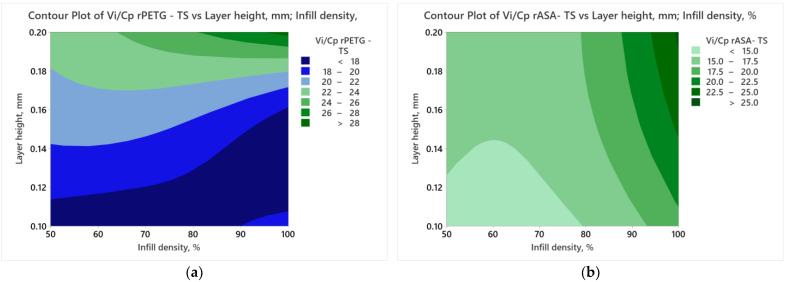
Contour plots charts for tensile strength: (**a**) rPETG; (**b**) rASA.

Figure 4 displays the contour plots obtained using the results of the *V_i_/C_p_* ratios of the additively manufactured samples of rPETG and rASA and the variable FDM parameters detailed in Table 1.

Analyzing Figure 4a, increasing both FDM parameters (L_h_ and I_d_) simultaneously results in higher *V_i_*/*C_p_* ratios, attributed to the lower production cost associated with the samples manufactured at maximum layer height. Figure 4b, reveals that the highest values of the *V_i_*/*C_p_* for tensile samples manufactured from rASA are achieved by utilizing a 100% filling percentage (I_d_ = 100%) and a layer height of 0.20 mm (L_h_ = 0.20 mm).

#### 3.1.2. Compressive Testing

Table 7 and Table 8 summarize the results obtained by applying relation 1 and calculating the production cost for compression samples fabricated through FDM using 100% recycled material from rPETG and rASA. Additionally, Table 9 and Table 10 present the V_i_/C_p_ results for compression samples manufactured via FDM from rPETG and rASA.

**Figure 5:**
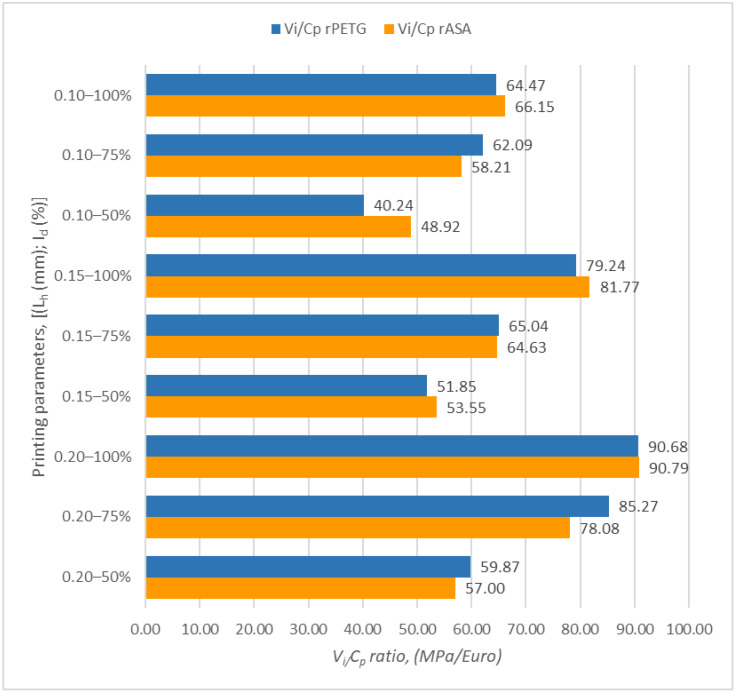
Ratio determination *V_i_/C_p_* for compressive samples made from rPETG and rASA.

Figure 5 shows the graphical representation of the ratios between V_i_ (compressive strength) and C_p_ (cost of production) of the samples additively manufactured by FDM using rPETG and rASA.

Analyzing the bar chart from Figure 5, it can be observed that the maximum value (90.79 MPa/EUR) of the ratio between V_i_ (compressive strength) and C_p_ (cost of production) was obtained for the set of additively manufactured rASA samples with the maximum layer height, L_h_ = 0.20 mm, and a maximum infill density percentage, I_d_ = 100%. The peak value of the report between V_i_ (compressive strength) and C_p_ (cost of production) of additively manufactured rPETG samples was obtained for samples manufactured with the parameters: L_h_ = 0.20 mm samples I_d_ = 100%.

Comparing the results between minimum and maximum values of the *V_i_/C_p_* ratios obtained for the rASA samples with those corresponding to the rPETG samples, it is found that the V_i_/C_p_ ratios of the rASA samples are higher by 0.12–17.75% compared to those of the rPETG FDM additively manufactured samples.

**Figure 6:**
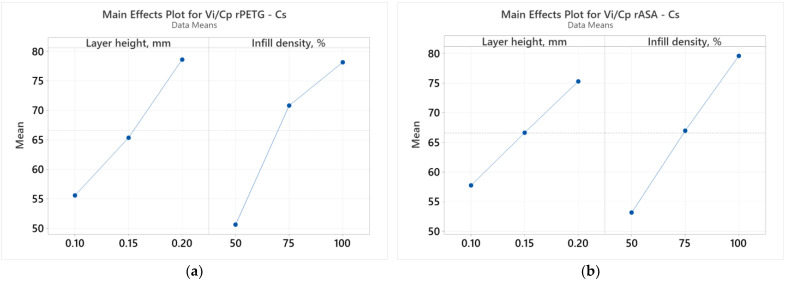
Main effects plots for compressive strength: (**a**) rPETG; (**b**) rASA.

Figure 6 displays the results of the ANOVA analysis examining the relationship between the FDM parameters (L_h_ and I_d_) and the results of the ratios between the compressive strength (V_i_) and the production cost (C_p_).

**Figure 7:**
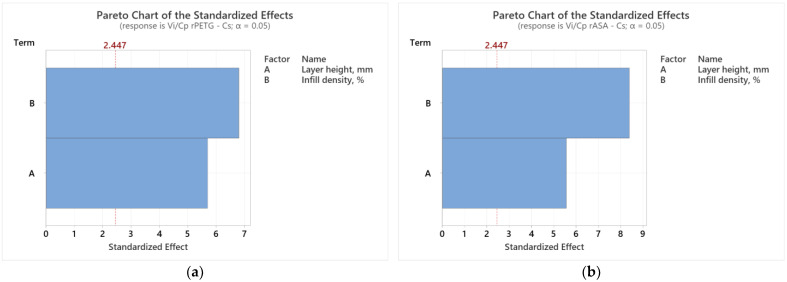
Pareto charts for compression strength: (**a**) rPETG; (**b**) rASA.

Analyzing the graphs presented in Figure 6, it is observed that the variable FDM parameters (L_h_ and I_d_) significantly affect the results of the *V_i_/C_p_* ratios of compression samples additively manufactured from rPETG (Figure 6a) and rASA (Figure 6b). According to Figure 6a, both FDM parameters have a significant influence on the results of the *V_i_*/*C_p_* ratios of compression samples manufactured from rPETG; however, the parameter with a greater influence is the infill density percentage (I_d_). Studying the graphs from Figure 6b, we observe that the considered FDM parameters (L_h_ and I_d_) significantly influence the results of the *V_i_*/*C_p_* ratios of compression samples additively manufactured from rASA, but the infill density percentage (I_d_) is the parameter with biggest influence on the results of the *V_i_*/*C_p_* ratios. The same conclusions are drawn from the analysis of the Pareto charts presented in Figure 7.

**Figure 8:**
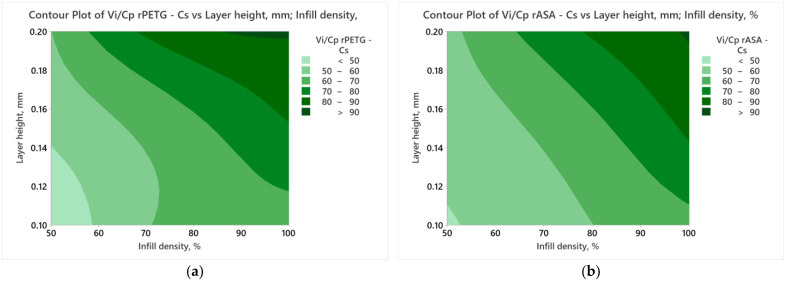
Contour plots charts for compression strength: (**a**) rPETG; (**b**) rASA.

Figure 8 presents contour plots obtained using the results of the *V_i_/C_p_* ratios of the additively manufactured samples of rPETG and rASA and the variable FDM parameters presented in Table 1.

Following the analysis of Figure 8, it can be concluded that the ratios between V_i_ and C_p_ are significantly influenced by the higher values of L_h_ and I_d_.

### 3.2. Optimization of FDM Parameters Based on Value Analysis to Improve 3D Printing Efficiency for Samples Made of rPETG and rASA

For technical–economic optimization, we utilized the Minitab 19 software, the variable parameters of the FDM as presented in Table 1, and fundamental value analysis to maximize the *V_i_/C_p_* ratio.

The optimization objective is to maximize of the results of the ratios between *V_i_*/*C_p_* for both mechanical tests performed (tension and compression) and both materials used (rPETG and rASA). Table 11 presents the targets for achieving optimization.

Using Relations (3) and (4) the desirability calculation was performed [3], written as follows:

(3) $$D={\left(d_{1}∙d_{2}∙\ldots {\;∙\;d}_{n}\right)}^{\frac{1}{n}}$$

(4) $$|d_{i}=0,\;if\;y_{i\;}<L_{i}\;d_{i}=\frac{\left(y_{i}-L_{i}\right)∙r_{i}}{\left(T_{i}-L_{i}\right)},\;if\;L_{i}\leq y_{i\;}\leq T_{i}\;d_{i}=1,\;if\;y_{i\;}>T_{i}$$

where D is the composite desirability; *n* is the number of responses; $d_{i}$ represents the desirability for each individual response; and $y_{i},\;L_{i}$ and $T_{i}$ represent the predicted value, target value, and lowest value, respectively, of the analyzed response of response.

Table 12 presents the overall desirability values for the printing parameters corresponding to each material type studied.

**Figure 9:**
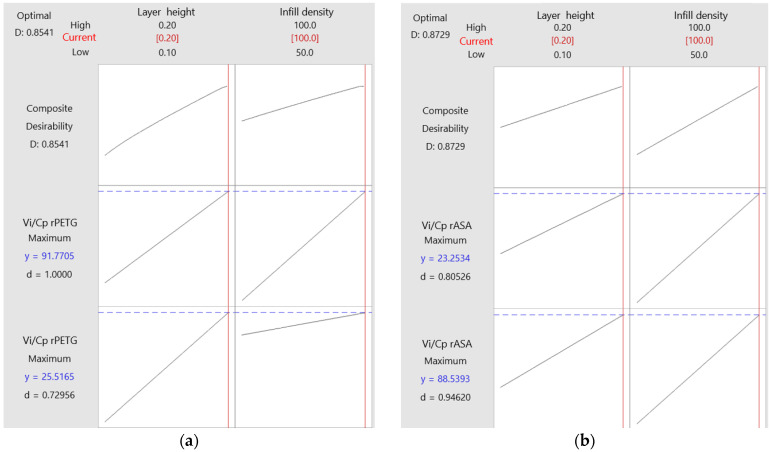
Optimization plots for 3D-printed materials: (**a**) rPETG; (**b**) rASA.

The graphs shown in Figure 9 illustrate the optimization of FDM variable parameters for producing rPETG and rASA samples [53].

The optimization graphs for FDM printing parameters related to the additive manufacturing of tensile and compression samples made from rPETG and rASA are illustrated in Figure 9.

The solid vertical red lines indicate the current arrangement of the factors, and the red numbers presented on each column reflect their current levels. The dashed horizontal blue lines emphasize the responses linked to the current settings of the factors, while the blue numerical values represent the responses related to these settings.

Based on the data from Figure 9a, the parameters for Fused Deposition Modeling (FDM) with rPETG are as follows: L_h_ = 0.20 mm and I_d_ = 100%. In Figure 9b, it can be seen that the optimization of the FDM parameters for rASA yielded these optimal settings: L_h_ = 0.20 mm and I_d_ = 100%.

## 4. Conclusions

This paper presents the results of a technical–economic study on the optimization of FDM parameters for manufacturing samples using recycled materials from PETG and ASA in the context of transitioning to a circular economy. To achieve this goal, we performed a multi-objective optimization with the aim of finding the optimal FDM parameters (L_h_—layer height and I_d_—infill density percentage) for the manufacture of parts from rPETG and rASA Everfill brand with 100% recycled material. By applying the fundamental principle of value analysis, which focused on maximizing the V_i_/C_p_ ratios, we used experimental data on tensile and compressive properties of additively manufactured rPETG and rASA samples, as well as production cost calculations, to optimize the FDM parameters for achieving the highest value in balancing mechanical characteristics and production cost.

Results from ANOVA analysis show that both FDM parameters considered (L_h_—the height layer and I_d_—the infill density percentage) significantly impact the *V_i_/C_p_* ratios. In the case of tensile samples additively manufactured from rPETG, the parameter that significantly influences the results of the *V_i_/C_p_* ratios is L_h_—the layer height, and in the case of compression samples manufactured from rPETG, the parameter that significantly influences the results of the *V_i_/C_p_* ratios is I_d_—the infill density percentage.

In the case of samples additively manufactured by FDM from rASA, the parameter that decisively influences the results of the *V_i_/C_p_* ratios of the tensile and compression samples is Id—the infill density percentage.

After optimizing the FDM process parameters, we found the following optimal parameters: L_h_ = 0.20 mm and I_d_ = 100%.

The results of this study demonstrate that the use of recycled materials for the manufacture of parts by FDM represents a good technical and economic solution, with the price of recycled materials being significantly lower compared to that of virgin materials. By applying the optimal printing settings determined in this study, reduced production costs can be achieved without impacting quality and mechanical characteristics.

The authors aim to increase the lifespan of the materials used in this study by reusing the two materials and determining the number of reuses until a significant decrease in mechanical characteristics is observed. In order to achieve a holistic analysis, in a future paper, we will conduct a study that will analyze the impact of 3D printing parameters (layer height, infill density, print speed, and extrusion temperature) on the mechanical characteristics of the leathers. Additionally, a comparison will be made between the theoretical material and energy consumption (determined by the slicer) and the material and energy consumption determined from measurements.

## Figures and Tables

**Table 1:** FDM printing parameters used to manufacture tensile and compressive samples from rPETG and rASA.

Printing Parameters	rPETG	rASA
Part orientation, P_o_	X-Y	X-Y
Extruder temperature, E_t_	250 °C	240 °C
Platform temperature, P_t_	70 °C	90 °C
Printing speed, P_s_	30 mm/s	30 mm/s
Infill pattern, I_p_	Grid	Grid
Layer height, L_h_	0.10; 0.15; 0.20 mm	0.10; 0.15; 0.20 mm
Infill density, I_d_	50; 75; 100%	50; 75; 100%
Plate adhesion, P_a_	Brim	Brim

**Table 2:** Testing conditions and samples dimensions for experimental investigation.

Mechanical Test	Testing Condition	Sample Dimensions
Tensile	- Barrus White 20 kN universal testing machine; - speed: 5 mm/min; - ambient temperature: 20 °C; - humidity: 40%.	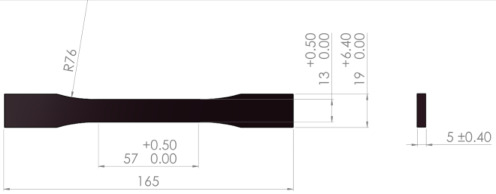
Compression	- Barrus White 20 kN universal testing machine; - speed: 10 mm/min; - ambient temperature: 20 °C - humidity: 40%	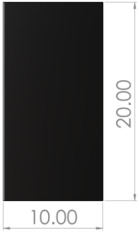

**Table 3:** Cost calculation for rPETG samples used for tensile testing.

Sample Set	L_h_ (mm)	I_d_ (%)	$C_{mat}$ (EUR)	$C_{en}$ (EUR)	$C_{p}$ (EUR)
1	0.10	100%	0.89	1.06	1.95
2		75%	0.77	0.73	1.50
3		50%	0.65	0.64	1.30
4	0.15	100%	0.89	0.67	1.56
5		75%	0.77	0.51	1.28
6		50%	0.65	0.49	1.14
7	0.20	100%	0.89	0.54	1.43
8		75%	0.77	0.38	1.15
9		50%	0.65	0.32	0.98

**Table 4:** Cost calculation for rASA samples used for tensile testing.

Sample Set	L_h_ (mm)	I_d_ (%)	$C_{mat}$ (EUR)	$C_{en}$ (EUR)	$C_{p}$ (EUR)
1	0.10	100%	0.94	1.06	2.00
2		75%	0.81	0.73	1.54
3		50%	0.69	0.64	1.33
4	0.15	100%	0.94	0.67	1.61
5		75%	0.81	0.51	1.32
6		50%	0.69	0.49	1.18
7	0.20	100%	0.94	0.54	1.48
8		75%	0.81	0.38	1.19
9		50%	0.69	0.32	1.01

**Table 5:** Ratio determination *V_i_/C_p_* for tensile samples made from rPETG.

Sample Set	Ultimate Tensile Strength (MPa)	$C_{p}$ (EUR)	*V_i_/C_p_* (MPa/EUR)
1	37.91	1.95	19.42
2	25.10	1.50	16.75
3	21.86	1.30	16.87
4	25.84	1.56	16.56
5	25.55	1.28	19.97
6	23.39	1.14	20.46
7	41.29	1.43	28.84
8	29.18	1.15	25.28
9	22.15	0.98	22.65

**Table 6:** Ratio determination *V_i_/C_p_* for tensile samples made from rASA.

Sample Set	Ultimate Tensile Strength (MPa)	$C_{p}$ (EUR)	*V_i_/C_p_* (MPa/EUR)
1	38.42	2.00	19.23
2	22.31	1.54	14.51
3	19.04	1.33	14.33
4	36.61	1.61	22.81
5	21.31	1.32	16.16
6	18.41	1.18	15.64
7	37.53	1.48	25.41
8	19.97	1.19	16.74
9	17.29	1.01	17.10

**Table 7:** Cost calculation for rPETG samples used for compressive testing.

Sample Set	L_h_ (mm)	I_d_ (%)	$C_{mat}$ (EUR)	$C_{en}$ (EUR)	$C_{p}$ (EUR)
1	0.10	100%	0.20	0.33	0.52
2		75%	0.20	0.23	0.42
3		50%	0.20	0.18	0.38
4	0.15	100%	0.20	0.22	0.42
5		75%	0.20	0.20	0.40
6		50%	0.20	0.12	0.32
7	0.20	100%	0.20	0.17	0.37
8		75%	0.20	0.11	0.31
9		50%	0.20	0.10	0.30

**Table 8:** Cost calculation for rASA samples used for compressive testing.

Sample Set	L_h_ (mm)	I_d_ (%)	$C_{mat}$ (EUR)	$C_{en}$ (EUR)	$C_{p}$ (EUR)
1	0.10	100%	0.21	0.33	0.53
2		75%	0.21	0.23	0.43
3		50%	0.21	0.18	0.39
4	0.15	100%	0.21	0.22	0.43
5		75%	0.21	0.20	0.41
6		50%	0.21	0.12	0.33
7	0.20	100%	0.21	0.17	0.38
8		75%	0.21	0.11	0.32
9		50%	0.21	0.10	0.31

**Table 9:** Ratio determination *V_i_/C_p_* for compressive samples made from rPETG.

Sample Set	Compressive Strength (MPa)	$C_{p}$ (EUR)	*V_i_/C_p_* (MPa/EUR)
1	33.83	0.52	64.47
2	26.28	0.42	62.09
3	15.22	0.38	40.24
4	33.10	0.42	79.24
5	26.07	0.40	65.04
6	16.69	0.32	51.85
7	33.28	0.37	90.68
8	26.49	0.31	85.27
9	17.92	0.30	59.87

**Table 10:** Ratio determination *V_i_/C_p_* for compressive samples made from rASA.

Sample Set	Compressive Strength (MPa)	$C_{p}$ (EUR)	*V_i_/C_p_* (MPa/EUR)
1	35.37	0.53	66.15
2	25.22	0.43	58.21
3	19.00	0.39	48.92
4	34.97	0.43	81.77
5	26.55	0.41	64.63
6	17.77	0.33	53.55
7	34.23	0.38	90.79
8	25.04	0.32	78.08
9	17.63	0.31	57.00

**Table 11:** Optimization goals for analyzed materials (rPETG and rASA).

Response	Goal	Lower		Target		Weight	Importance
*Vi/Cp*		rPETG	rASA	rPETG	rASA		
Tensile [MPa/EUR]	Maximum	16.56	14.33	28.84	25.41	1	1
Compression [MPa/EUR]		40.24	48.92	90.68	90.79		

**Table 12:** Composite desirability.

Printing Parameters		Material	
Layer Height, (mm)	Infill Density, (%)	rPETG	rASA
		Composite Desirability	Composite Desirability
0.10	100	0.219161	0.497296
	75	0.075384	0.185597
	50	0.000000	0.000000
0.15	100	0.568485	0.685130
	75	0.422005	0.373285
	50	0.262126	0.050715
0.20	100	0.854140	0.872890
	75	0.705339	0.560955
	50	0.534216	0.246367

## Data Availability

Data are contained within the article.
